# Cancer Diagnostic and Predictive Biomarkers

**DOI:** 10.1155/2014/980163

**Published:** 2014-04-03

**Authors:** Franco M. Buonaguro, David Pauza, Maria Lina Tornesello, Pierre Hainaut, Renato Franco, Francesco M. Marincola

**Affiliations:** ^1^Department of Experimental Oncology, Istituto Nazionale Tumori IRCCS Fondazione Pascale, 80131 Napoli, Italy; ^2^Department of Microbiology and Immunology, Institute of Human Virology, Baltimore, MD 21201, USA; ^3^Department of Research, International Prevention Research Institute, 69006 Lyon, France; ^4^Department of Pathology, Istituto Nazionale Tumori IRCCS Fondazione Pascale, 80131 Napoli, Italy; ^5^Department of Research, Sidra Medical and Research Center, 00974 Doha, Qatar

## 1. Introduction

The new high-throughput “omics” technologies have recently opened the possibility to identify molecular changes and metabolic pathways in each cancer type with the possibility of molecular tumor subclassification and identification of a more reliable prognosis and appropriate treatment [1–3]. A further result is the reduction of overtreatment of those with higher responsivity and better prognosis. Furthermore, such studies do have the possibility of identifying specific cancer targets, with higher effectiveness of chemotherapy and much lower general toxicity. The whole field is in a current turmoil, and this specific issue, focused on the clinical validation of newly identified biomarkers, has been able to bridge molecular mechanisms with clinical diagnosis and therapeutic responsivity touching different aspects of this topic.

The two major topics have been (a) characterization of biomarkers for more specific cancer diagnosis and prognosis; (b) identification of biomarkers for progression evaluation and therapeutic responsivity. Biomarkers of cancer susceptibility have been also reported.

## 2. Diagnostic and Prognostic Biomarkers

Nowadays, diagnosis of cancer is still mainly based on morphological features evaluated by histological analyses. Although this approach leads to a confident diagnosis in most cases, the molecular characterization of the cancer tissues can contribute to better establish the tumor grade and aggressiveness, as well as to predict the possible outcomes for the different available treatments. The molecular characterization will become an invaluable tool for clinicians in the decision-making process. In this respect T. Sequeiros et al. describe the new opportunities offered by the analysis of differentially expressed miRNAs and proteins in distinguishing between normal and malignant prostate tissues. Thus, miRNA and protein expression profiles can be used to correctly classify even poorly differentiated prostate tumor samples, which cannot be clearly diagnosed with currently available techniques.

M. L. Tornesello et al. describe clinically validated or new candidate viral and cellular biomarkers which can be useful for the diagnosis of cervical intraepithelial lesion at high risk of progression. In particular the authors focused on specific assays, such as HPV DNA, HPV E6/E7 mRNA, HPV proteins, p16(INK4a) and Ki67, TOP2A, and MCM2 cellular factors as well as DNA methylation profiles, and their improved sensitivity and specificity in identifying premalignant lesions at high risk of evolving into invasive cervical cancer.

A further aspect is the identification of markers for early diagnosis, in order to perform radical treatment with the less invalidating procedures. G. Aquino et al. show that expression of SPARC/Osteonectin in oral squamous cell carcinoma (OSCC) represents a good prognostic marker with a significant statistical correlation between the expression of SPARC in the tumor and a better overall survival (*P* < 0.034). The protein, whose expression correlates with exposure to alcohol and cigarette smoking, however, because of its presence in the deep side of the tumor, is not well detected in biological samples, such as scraping and saliva. Further biomarkers are requested for noninvasive population screenings. On the contrary expression of Beta-catenin was a negative prognostic marker. A. Santoro et al. report their observation based on 374 oropharyngeal cancers. Beta-catenin protein was mainly detected in the cytoplasm of cancerous cells and only focal nuclear positivity was observed. High cytoplasmic expression correlated significantly with poor histological differentiation, advanced stage, and worst patient outcome (*P* < 0.05), supporting a more specific and aggressive treatment.

A peculiar place is occupied by potential markers which are differentially expressed in different types of cancers. For such markers it is extremely relevant to clearly define the expression levels in cancer subtypes and elaborate specific ranges. A. Borrelli et al. describe as paradigm the manganese superoxide dismutase (MnSOD), which is overexpressed in gastric and esophageal, lung, and colorectal cancers. The high expression levels of MnSOD in those cancers are associated with the aggressiveness of cancer and its metastatic potential, along with a poor prognosis. In glandular cancers, instead, MnSOD expression is mainly inversely correlated with cancer cell growth, being lower in aggressive breast, pancreatic, and ovarian cancer. Moreover, reestablishment of normal MnSOD levels in tissues seems to play a relevant role for their radiosensitivity, acting as radiosensitizer for cancer tissues and protecting the adjacent normal tissue.

Glycosylation is a posttranslational modification of proteins playing a major role in cell signaling, immune recognition, and cell-to-cell interaction. F. M. Tuccillo et al. review the aberrant protein glycosylation associated with human cancer and the identification of protein glycoforms as cancer biomarkers. In particular, they describe aberrant CD43 glycosylation as cancer biomarker and the identification of UN1 monoclonal antibody (UN1 mAb) able to recognize aberrant CD43 glycoforms associated with human cancers. UN1/CD43 glycoforms have been detected especially in breast cancer cells, where their expression levels are directly correlated with the progression stage of the disease.

F. Morandi et al. showed that low levels of soluble HLA-E and -F are significantly associated with worse event-free survival and overall survival in the whole cohort of neuroblastoma patients and in those with metastasis. J. Polivka et al. report that mutations in metabolic enzyme IDH, isocitrate dehydrogenases (IDH1 and IDH2), represent relevant prognostic biomarkers in glioblastoma multiforme (GBM), the most malignant primary brain tumor in adults. The IDH1 R132H mutation is mainly mutated in secondary GBMs (89.9%), and it is observed with low frequency in primary GBMs (15.3%). IDH1 mutation is a good prognostic biomarker given that patients with mutation had significantly longer PFS and OS than patients with wild-type IDH1 and suffered more likely from secondary GBMs.

Among the most effective biomarkers, oncofetal biomarkers are the most peculiar and the most sensitive (i.e., alpha-fetoprotein) being expressed in early developmental stages and in advanced cancers. Moreover, such markers support the hypothesis that cancer cells properties are somehow related to undifferentiated status. D. E. Polev et al. describe the identification of a new biomarker associated with the* ELFN1* gene, abundantly expressed in tumors (and in fetal brain) but weakly expressed in few normal tissues. The secondary structure of the major transcript variant revealed a hairpin-like structure characteristic of precursor microRNAs. Moreover by comparative genomic analysis the authors show that the gene originated* de novo *in primates and describe a region responsive to c-MYc, suggesting its relevant role in carcinogenesis and a potential role as diagnostic/prognostic cancer biomarker.

## 3. Predictive and Responsive Biomarkers

Responsivity to hormonal therapy as well as chemotherapy is highly variable among cancer patients and such variability is not only related to the cancer TNM stage but also to other factors which still need to be fully elucidated. In this context S.-H. Kuo et al. describe the relevance of determining the polymorphism of CYP19 gene. The AASA haplotype in hormone receptor-positive breast cancer patients, with lymph node-negative, is associated with poor survival of premenopausal women but does not affect survival of postmenopausal women.

A peculiar application of molecular techniques to evaluate therapeutic responsivity is the presence of EGFR mutations in non-small-cell lung cancers (NSCLC). Several studies, indeed, have shown that NSCLC patients carrying EGFR mutations significantly benefit from first-line therapy with specific tyrosine kinase inhibitors TKIs [2]. C. Roma et al., in this special issue, describe the more sensitive and specific technique currently available to choose the most active treatment in NSCLC patients.

Genetic polymorphisms of the bone morphogenic proteins 4 (BMP-4) have been associated with the response to platinum-based chemotherapy and the clinical outcome in patients with advanced NSCLC. T. Zhennan et al. show that the variant genotypes of 6007 C > T polymorphisms are significantly associated with the chemotherapy response. The CC genotype carriers have a 2.77 higher chance to be responder to chemotherapy (*P* < 0.001). Moreover patients with high BMP-4 expression had a 2.81 higher chance to be resistant to chemotherapy than those with low BMP-4 expression (*P* = 0.01) and the hazard ratio (HR) for 6007TT was 2.37 time higher than 6007CC (*P* = 0.003). These results suggest that SNPs and tissue expression of the* BMP*-4 gene are potential predictor for the chemotherapy response and prognosis of advanced NSCLC.

M. Walentowicz-Sadlecka et al. describe the relevance of preoperative maximum standardized uptake value (SUVmax), measured by 18F-FDG PET/CT, as prognostic marker for primary endometrial cancers. The mean preoperative SUVmax was significantly lower for FIGO I than for higher stages. In particular analysis of survival ROC curve reveals that SUVmax values >17.7 predict high risk of recurrence and lower OS of endometrial cancer patients. For such reason the preoperative SUVmax is proposed as an important indicator reflecting tumor aggressiveness and predicting poor prognosis. It would be useful for making noninvasive diagnoses and deciding the appropriate therapeutic strategy for endometrial cancer patients.

Alterations of cyclins (CCNs) and cyclin-dependent kinases (CDKs) complexes and degradation of CCNs have been reported in more than 90% of human cancers, and the most frequent are those related to the G1 phase. P. Bonelli et al. correlate alterations of cell cycle regulators with human cancers and therapeutic responsivity. In particular CCNE overexpression correlates with aggressiveness of breast cancer as well as with gastric cancer progression, predicting risk of distant recurrence in the abdomen. Also loss of* CDKN2A*,* CDKN1B*, and* CDKN1A* is predictor of poor prognosis in several types of cancer, and codeletion of* CDKN2B/CDKN2A *genes is significantly related to a negative prognosis of NSCLC and ALL patients. Moreover overexpression of CCND is associated with higher chemotherapy resistance and recurrence of head and neck cancers. Treatment with molecules inhibiting CCNs (including specific interfering miRNAs as well as nonsteroidal anti-inflammatory drugs—NSAID) inhibits the G1/S transition. The resulting G0/G1 arrest is associated with an increase of TP53 and CDKN1A, along with a downregulation of transcripts encoding enzymes involved in DNA precursor synthesis, DNA replication system, and DNA-repair mechanisms. As consequence, a prolonged high-dose ibuprofen treatment, resulting in upregulation of caspase transcripts and activation of an apoptotic program of cancer cells, could represent an effective anticancer strategy, particularly for local treatment.

## 4. Genetic Determinants as Susceptibility Biomarkers

A further set of biomarkers is represented by those able to identify patients at high risk of specific cancers. G. Ponti et al. describe missense versus nonsense PTCH1 mutations, associated with protein profiles specific in nevoid basal-cell carcinomas syndrome (NBCCS); in particular overexpression of the matrix metalloproteinases 1 (MMP1) has been reported in the fibroblast conditioned media of NBCCS patients.

Genetic determinants are also relevant for pathogen-driven diseases and risk of progression to cancer. V. De Re et al. report the strong correlation between IL28B C allele and spontaneous hepatitis C virus (HCV) elimination. The IL28B TT genotype, instead, is associated with persistent chronic hepatitis which leads to both hepatocyte injury and chronic inflammation, promoting HCC development. Patients with lymphoproliferative disorders, like all chronic HCV-related diseases, showed a lower CC frequency than patients who spontaneously eliminate the virus, without a significant difference for IL28B rs1297860 allelic distribution as compared to chronic HCV patients. Specific human SNPs are becoming relevant biomarkers of persistent infections and progression risk of cancers.

Our major aim in organizing this volume was to emphasize the need for noninvasive diagnostic and prognostic markers for a more accurate identification and staging of cancer lesions in order to have a better prognostic evaluation of cancer patients. Moreover the identification and the use of predictive biomarkers would allow the optimal implementation of specific therapeutic protocols, tailored to individual patient or specific classes of patients. Finally, cancer susceptibility biomarkers could have the critical role to limit carcinogenic, genotoxic exposure to such individuals in order to strongly reduce their cancer progression risk.

We hope that this special issue can contribute to this scientific area, bringing to readers accurate data and relevant features of the several discussed cancer biomarkers, but, mainly, we hope that this special issue will initiate new discussions relating to the identification of more sensitive and specific diagnostic, prognostic, and therapeutic predictive biomarkers.

*Franco M. Buonaguro* *David Pauza* *Maria Lina Tornesello* *Pierre Hainaut* *Renato Franco* *Francesco M. Marincola*
